# miR-365 inhibits duck myoblast proliferation by targeting IGF-I via PI3K/Akt pathway

**DOI:** 10.1042/BSR20190295

**Published:** 2019-11-15

**Authors:** Wenqiang Sun, Shenqiang Hu, Jiwei Hu, Shuang Yang, Bo Hu, Jiamin Qiu, Xiang Gan, Hehe Liu, Liang Li, Jiwen Wang

**Affiliations:** Farm Animal Genetic Resources Exploration and Innovation Key Laboratory of Sichuan Province, Sichuan Agricultural University, Ya’an, Sichuan 625014, P.R. China

**Keywords:** Duck, IGF-I, miR-365, Myoblast proliferation, PI3K/Akt pathway

## Abstract

miR-365 is found to be involved in cancer cell proliferation and apoptosis. However, it remains unknown if and how miR-365 plays a role in myoblast proliferation. In the present study, we found that overexpression of miR-365 can inhibit duck myoblast proliferation. To uncover the mechanism by which miR-365 inhibits duck myoblast proliferation, we showed that miR-365 can down-regulate insulin-like growth factor-I (IGF-I) by directly targeting its 3′untranslated region (UTR). Moreover, enhanced miR-365 decreased the mRNA expression of PI3K, Akt, mTOR and S6K. Importantly, the enhanced PI3K, Akt, mTOR and S6K expression by miR-365 inhibitor (anti-miR-365) was abrogated by treatment with LY294002, a PI3K inhibitor. Together, our results indicated that miR-365 may target IGF-I to inhibit duck myoblast proliferation via PI3K/Akt pathway.

## Introduction

Skeletal muscle development (myogenesis) is a multistep process that commences with the commitment of multipotent precursor cells to myoblasts, followed by proliferation, withdrawal from the cell cycle, differentiation and fusion into multinuclear myotubes and then myofibers [1,2]. During the highly orchestrated process, myoblast proliferation is an early cellular event critical for myogenesis, which is controlled by a multitude of signaling cascades initiated by various autocrine/paracrine growth factors and cytokines [3].

MicroRNAs (miRNAs), 18–25 nucleotides, small single stranded non-coding RNAs, negatively regulate gene expression by binding to the 3′untranslated regions (UTRs) of target mRNAs [4]. It is well known that miRNAs are involved in many biological processes, including proliferation, differentiation and apoptosis [5]. Recently, lots of miRNAs have been characterized as modulators of myoblast proliferation and myogenic differentiation [6–10]. For example, miR-27 has been identified as a key regulator of Myostatin, a known inhibitor of myogenesis, which promotes myoblast proliferation [11]. miR-148 could promote myogenic differentiation by targeting the ROCK1 gene, a known inhibitor of myogenesis [12]. These findings suggest that miRNAs play critical roles in skeletal muscle development.

miR-365 is located on chromosome 16p13.12. It has been demonstrated that miR-365 is involved in cell proliferation and apoptosis in many kinds of cell. miR-365 could inhibit cell cycle progression and promote apoptosis of colon cancer cells [13]. miR-365 could potentiate ox-LDL-induced endothelial cells apoptosis by regulating expression of Bcl-2 [14]. miR-365 could also stimulate cell proliferation and differentiation by targeting histone deacetylase 4 (HDAC4) in chondrocytes [15]. Facioscapulohumeral muscular dystrophy (FSHD) is one of the most frequent muscular dystrophies. Previous study showed that miR-365 was up-regulated in the FSHD primary myoblasts compared with control [16]. This finding suggested that miR-365 is essential for myoblast development. However, a role of miR-365 in the regulation of skeletal muscle development has not been described.

In the present study, in order to determine the role of miR-365 in myoblast proliferation, the effect of miR-365 overexpression on cell proliferation activity was investigated. Moreover, to further explore the underlying molecular mechanism, the effect of miR-365 ectopic expression on the insulin-like growth factor-I (IGF-I)/PI3K/Akt and cell cycle was also investigated.

## Materials and methods

### Animals

For each experiment, eight hatching Peking duck eggs were obtained after 13 days of incubation from Sichuan Agricultural University Waterfowl Breeding Experimental Farm. All the experiments took place in the Sichuan Agricultural University. These eggs were selected by chance and were incubated under the same conditions at a temperature of 37 ± 0.5°C and humidity of 86–87%. The present study was carried out in strict accordance with the recommendations in the Guide for Sichuan Agricultural University Animal Care and Use Committee, Sichuan Agricultural University, Sichuan, China. This work was carried out with the ethics approval of Sichuan Agricultural University Animal Care and Use Committee.

### psi-CHECK-2 dual-luciferase reporter vector construction

The IGF-I 3′UTR sequence including the miRNA binding site was amplified using P1, a mutagen in the miR-365 binding site of IGF-I 3′UTR was generated with a pair of mutagenic primers P2. The cyclin D1 (CCND1) 3′UTR sequence including the miRNA binding site was amplified using P3, these fragments were ligated into the psi-CHECK-2 dual-luciferase reporter vector (Promega, U.S.A.) using restriction enzymes Xho I and Not I (TaKaRa, Japan) and then ligated by T_4_ DNA ligase (TaKaRa, Japan), respectively.

### Isolation and culture of duck myoblasts

For each experiment, Peking duck eggs which were incubated for 13 days were randomly selected, and the duck myoblasts that used were isolated from the leg muscles of embryos [17]. Cells were cultured in growth medium (GM) containing Dulbecco’s modified Eagle’s medium (DMEM) (Tokyo, Japan), 15% fetal bovine serum (FBS) (Invitrogen, U.S.A.) and antibiotics (100 U/ml penicillin and 100 g/ml streptomycin). Growing myoblasts (70–80% confluent) were transfected with miR-365 mimic (50 nM) by using Lipofectin 2000 (Invitrogen, U.S.A.) according to the manufacturer’s instructions. At 24 h post-transfection, cells were harvested for RNA and protein extraction.

### Cell viability analysis

Duck myoblasts were seeded at a density of 5 × 10^3^ cells/well in a 96-well plate, and cell viability was analyzed by cell counting kit-8 (CCK-8) (Bestbio Biotechnology, China). After transfection or LY294002 treatment, the cells were incubated with CCK-8 for an additional 4 h at 37°C until an orange-colored product was yielded. The degree of the color was directly proportional to the number of viable cells. The absorbance at 450 nm was measured using a microplate reader (Thermo, U.S.A.). The samples from each treatment at each time point had six replicates.

### BrdU assay and immunofluorescence

For the proliferation assays, myoblasts were incubated with 25 μM of 5-bromo-2V-deoxyuridine (BrdU) (10 mg/ml in PBS, Boster, Beijing, China) for 4 h at 37°C in the incubator. Immunofluorescence labeling was performed according to the method previously described by Liu et al. [19]. Briefly, each well was washed three times with PBS to remove the culture medium. Then, the myoblasts were fixed with paraformaldehyde solution (4%) and were treated with Triton-100 solution (0.05%) in PBS for 20 min. Blocking was conducted using a blocking solution (1% bovine serum albumin in PBS) for 30 min, and the anti-BrdU antibody (antibody was diluted 1:20 with PBS; Solarbio Co., Beijing, China) was added to the wells and incubated overnight at 4°C. Then, the cells were washed three times with PBS and incubated with a goat anti-mouse IgG antibody (antibody was diluted 1:200 with PBS; Boster, China) at 37°C for 2 h. Then, the nuclei were labeled with DAPI (10 μg/ml in PBS; BiYunTian Biotechnology, China). Finally, the myoblasts were observed using a florescence microscope (Nikon, Germany), and the photos were analyzed using the Image-Pro Plus 6.0 software (Media Cybernetics, Bethesda, MD).

### Luciferase reporter assays

Duck myoblasts were transfected with miRNAs using Lipofectamine 2000 transfection reagent twice in a 24-h interval. Six hours after the last transfection, luciferase plasmids were transfected using Lipofectamine 2000. Control vector psi-CHECK-2 (Promega) was transfected as an internal control. At 48 h after plasmid transfection, luciferase assays were performed with the Dual-Luciferase reporter assay system (Promega) by following the manufacturer’s instructions. The luminescent signal was quantified with a luminometer (Monolight 3020; BD Biosciences). Each value from the *Renilla* luciferase construct (rr) was first normalized to the firefly (*Photinus pyralis*) luciferase value (pp) from the co-transfected psi-CHECK-2 control vector. Each rr/pp value in the miRNA transfections was again normalized to the rr/pp value obtained in control psi-CHECK-2-transfected cells.

Cells were cultured in 48-well plates when the cell growth reached approximately 80% confluence. The miR-365 mimic and psiCHECK-2- IGF-I-3′UTR (IGF-I-UTR-W), psi- CHECK-2- IGF-I-mut-3′UTR (IGF-I-UTR-Mut) or psiCHECK-2- CCND1-3′UTR (CCND1-UTR-W) were cotransfected into cells by Lipofectamine 2000. The transfection reagent was replaced with fresh growth medium (DMEM with 10% FBS) after transfection for 4–6 h. Next, the cells were washed with PBS and harvested using 200 ml Passive Lysis Buffer (PLB) and rocked for 30 min at room temperature. Dual-luciferase activity was measured by MPPC luminescence analyzer (HAMAMATSU; Beijing, China) and the *Renilla* luciferase activity was normalized against Firefly luciferase activity.

### Real-time PCR

Total RNA was extracted from myoblasts by TRIzol (Takara, Japan) according to the manufacturer’s instructions and then measured by spectrophotometer. RNA was reverse-transcribed to synthesis the cDNA by using the reverse transcript system (Takara, Japan). Real-time PCR (RT-PCR) was carried out with SYBR Prime Script RT-PCR Kit (TaKaRa, Japan) using the Bio-Rad CFX Manager (Bio-Rad Laboratories, U.S.A.). One sample collected from cells was repeated thrice. The relative expression of target genes was normalized against internal control gene which is duck *GAPDH*. Relative gene expression was analyzed by the comparative *C*_t_ method (2^−ΔΔ*C*_t_^ method) [18]. Primer sequences (P4–P13) were used for RT-PCR (Table 1). In order to determine the RT-PCR efficiency of target and internal control genes, ten-fold serial dilution (10^−1^–10^−5^) of cDNA were made and assayed in triplicate to produce standard curves. The identity of the amplified products was also confirmed by sequencing (Applied Invitrogen, China).

**Table 1:** Primer sequences used in the present study

Primer ID	Primer name	Primer sequence (5′–3′)
P1	Psi-IGF-I-F	CCGCTCGAGACCTGAGGAGGCTGGAGATGTACTG
	Psi-IGF-I-R	ATAAGAATGCGGCCGCGAATGTTTAGTTGCATTGTTCACTGGG
P2	Psi-IGF-I-MUT-F	AACCAATTTTACGGCTCCCAGT GAACAATGCAACTAAACATTCCAATATT
	Psi-IGF-I-MUT-R	ACTGGGAGCCGTAAAATTGGTTGAGATTGCATCAAGCTTTTTAACCAT
P3	Psi-CCND1-F	CCGCTCGAGTTCTTGCTCTGTCTCCCTTCCATCT
	Psi-CCND1-R	ATAAGAATGCGGCCGCAGCCTAGAACTGCGTTAAAGCTATGCT
P4	IGF-I-F	GTGAAGATGCATACTGTGTC
	IGF-I-R	TGAAGTAAAAGCCTCTGT
P5	PI3K-F	CTTTTACCGAGGAGGTTCTGTGG
	PI3K-R	CTGAAGGTTGGTCTTTGTGGAC
P6	Akt-F	TCTTTGCTGGCATTGTTTGGC
	Akt-R	GCTGTCATCTTGGTCAGGAGGAGT
P7	mTOR-F	CTATCTGCCTCAGCTCATTCCT
	mTOR-R	GTCATCCAGGTTAGCTCCAAAG
P8	p70S6K-F	ATAATCGTGCTGTGGACTGGTG
	p70S6K-R	TCTGGCTTCTTGTGTGAGGTAGG
P9	p21-F	TTGGCTCACAAGGTCCCATCTAAGG
	p21-R	TTGGCTCACAAGGTCCCATCTAAGG
P10	CCND1-F	CTGGATGCCAACCTCCTCAACG
	CCND1-R	GCACTTCTGCTCCTCGCAAACCT
P11	CCND2-F	ACAATCCCCCTCACAGCAGAGAAGC
	CCND2-R	TGCGAGGTTCCACTTCAACTTCCC
P12	CCND3-F	ACAGCACGAGGCGAGCAAGGAG
	CCND3-R	GGGACCGAGGACAGGTAGCGAT
P13	GADPH-F	AAGGCTGAGAATGGGAAAC
	GADPH-R	TTCAGGGACTTGTCATACTTC

F,R: forward and reverse primers, respectively. Ta Opt means annealing temperature.

### Western blot

Total cellular proteins were extracted from duck myoblasts with RIPA lysis buffer (Beyotime Biotech, China). Protein samples were resolved by 10% SDS/PAGE and electroblotted on polyvinylidene difluoride (PVDF) membranes (Beyotime Biotech, China). The membranes were incubated in block buffer (Beyotime Biotech, China) at 37°C for 2 h and then incubated with the primary antibody at 4°C for 12 h. After that, the membranes were incubated with a secondary antibody at 37°C for 2 h, and subsequently detected by using the ECL kit (Beyotime Biotech, China) and a Gel Imaging System (Bio-Rad, U.S.A.). Primary antibodies against the following proteins were purchased from Biosynthesis Biotechnology and used at 1:1000 dilutions: mTOR, phospho-mTOR, p70S6K, phospho-p70S6K (Ser^417^), Akt, phospho-Akt, while GAPDH (diluted 1:1000) was purchased from Beyotime Biotech. The secondary antibodies (HRP-conjugated goat anti-rabbit IgG or goat anti-mouse IgG) were purchased from Biosynthesis Biotechnology as well. ImageJ software (National Institutes of Health, U.S.A.) was used for densitometry analysis. The values below each Western blot image represent the relative abundance of the target protein compared with GAPDH.

### Statistical analysis

The data were subjected to analysis of variance (ANOVA) and the means were compared for significance by Tukey’s test. ANOVA and *t* tests were performed using SAS (SAS Institute, Cary, NC, U.S.A.) and the results were expressed as the mean ± S.D.

## Results

### miR-365 inhibited duck myoblast proliferation

**Figure 1:**
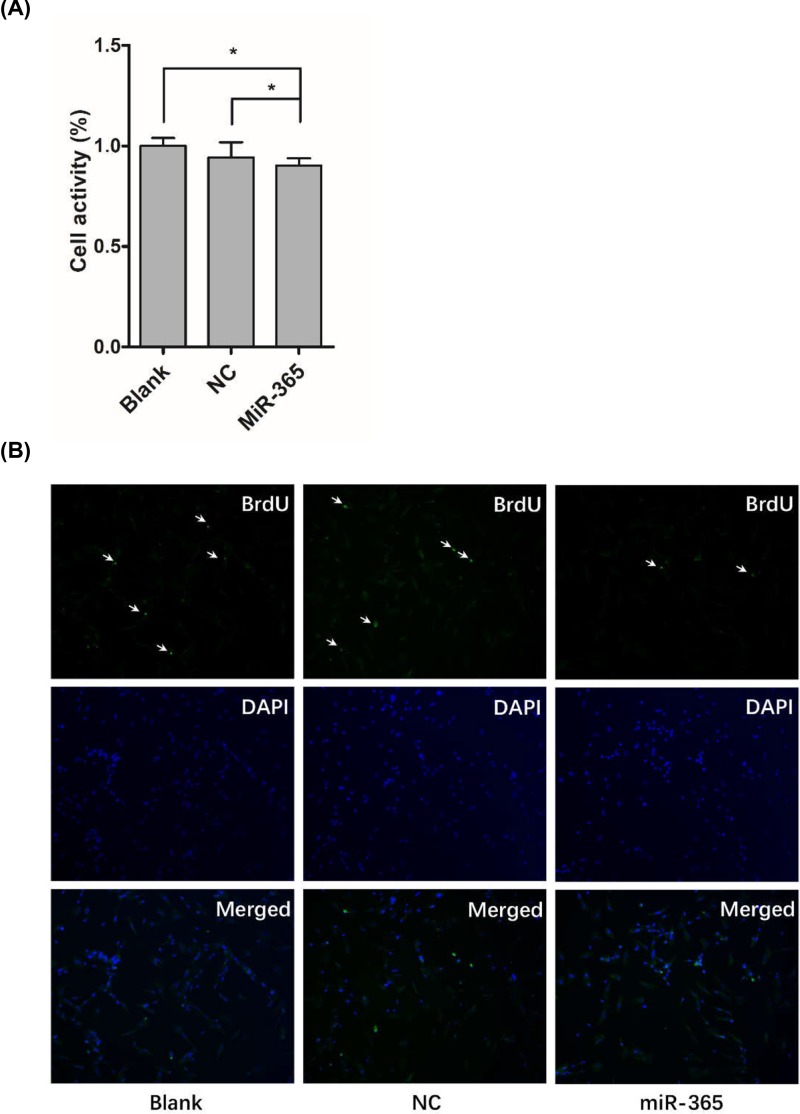
Influences of miR-365 overexpression on cell proliferation (**A**) CCK-8 assay was performed to detect the cell viability. (**B**) BrdU staining was performed to detect the myoblast number. Quantification of the positive BrdU cell (upper panel, green color) and normalized against the total number of nuclei (middle panel, purple color). Each point represents the relative mean ± SD. * denotes significance (*P*<0.05).

In order to explore the role of miR-365 in duck myoblast proliferation, primary duck myoblast was transiently transfected with negative control or miR-365 mimics. To determine the effect of miR-365 on cell proliferation, we performed the CCK-8 and BrdU incorporation assay and found that the duck myoblast viability was significantly inhibited by miR-365 (Figure 1A) (*P*<0.05). Moreover, BrdU staining result showed that the number of BrdU positive cells in miR-365 mimic transfection group was lower than two control group (Figure 1B). Together, these data suggest that miR-365 can inhibit duck myoblast proliferation.

### IGF-I was a direct target of miR-365 in duck

**Figure 2:**
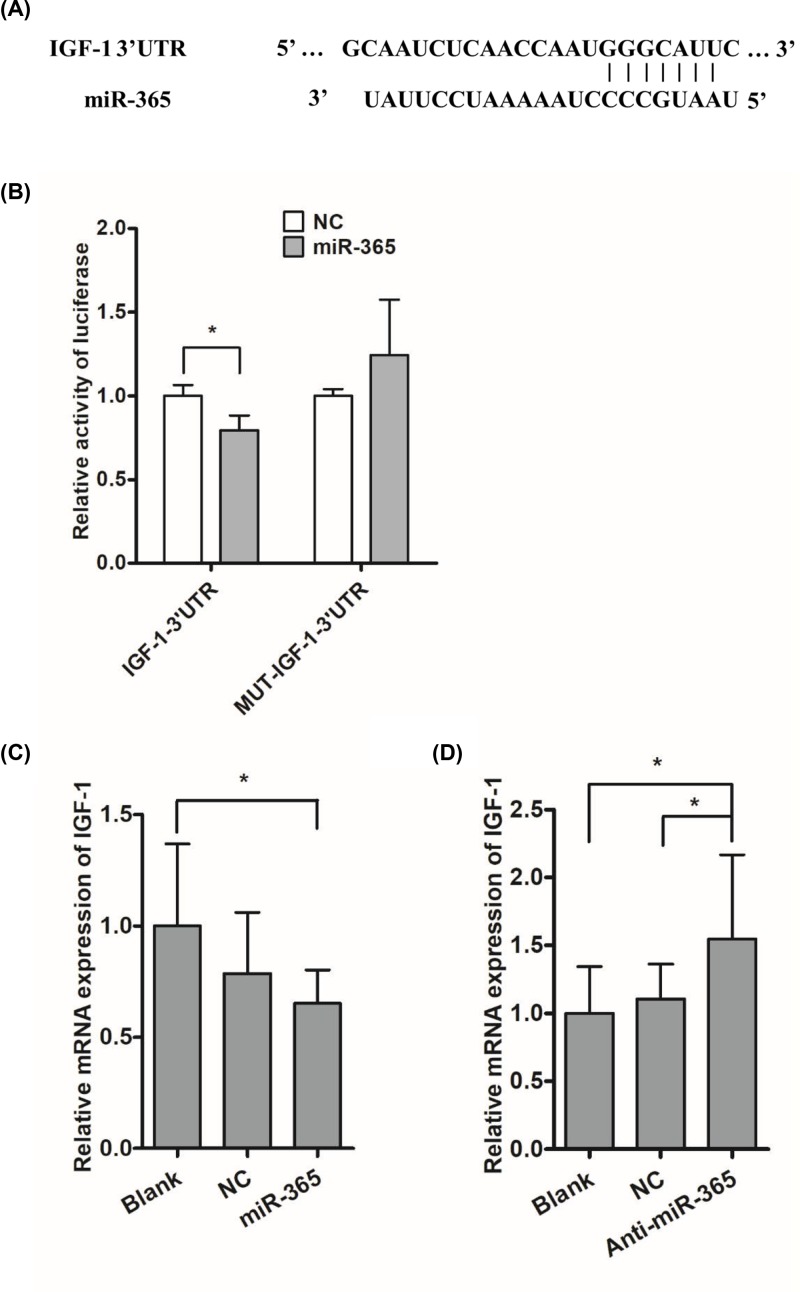
miR-365 down-regulates IGF-I by directly targeting its 3′UTR (**A**) miR-365 target sequence alignment in the IGF-I 3′UTR. (**B**) Activity of a luciferase reporter fused to IGF-I 3′UTR and IGF-I 3′UTR mutated fragments transfected into duck myoblast that were kept in growing DMEM. (**C**) Influences of miR-365 mimic overexpression on IGF-I expression. (**D**) Influences of anti-miR-365 overexpression on IGF-I expression. Each point represents the relative mean ± SD. * denotes significance (*P*<0.05).

Previously, IGF-I has been shown to promote myoblast proliferation and protect myoblast from apoptosis, suggested that it played a critical role in myoblast proliferation. Here, IGF-I was identified as a target gene of miR-365 by micro-RNA.org (http://www.microrna.org/microrna/), an online prediction tool for predicting target genes of miRNAs. The prediction tool revealed a high degree of conservation in the binding domain of 3′UTR of IGF-1 to miR-365 (Figure 2A). To verify this, the dual-luciferase reporters of IGF-I were co-transfected with miR-365 mimic or control into cells. We found that miR-365 significantly decreased the firefly luciferase activity of the wild-type IGF-I reporter compared with control group (Figure 2B). Furthermore, when the predicted miR-365 seed region in the 3′-UTR was mutated, the mutant reporter no longer responded to miR-365 (Figure 2B). Consistent with these data, we found the level of IGF-I transcript was down-regulated by miR-365 mimic (Figure 2C), while the level of IGF-I transcript was markedly up-regulated by anti-miR-365 (*P*<0.05) (Figure 2D).

### miR-365 inhibited the activation of PI3K/Akt/mTOR pathway

**Figure 3:**
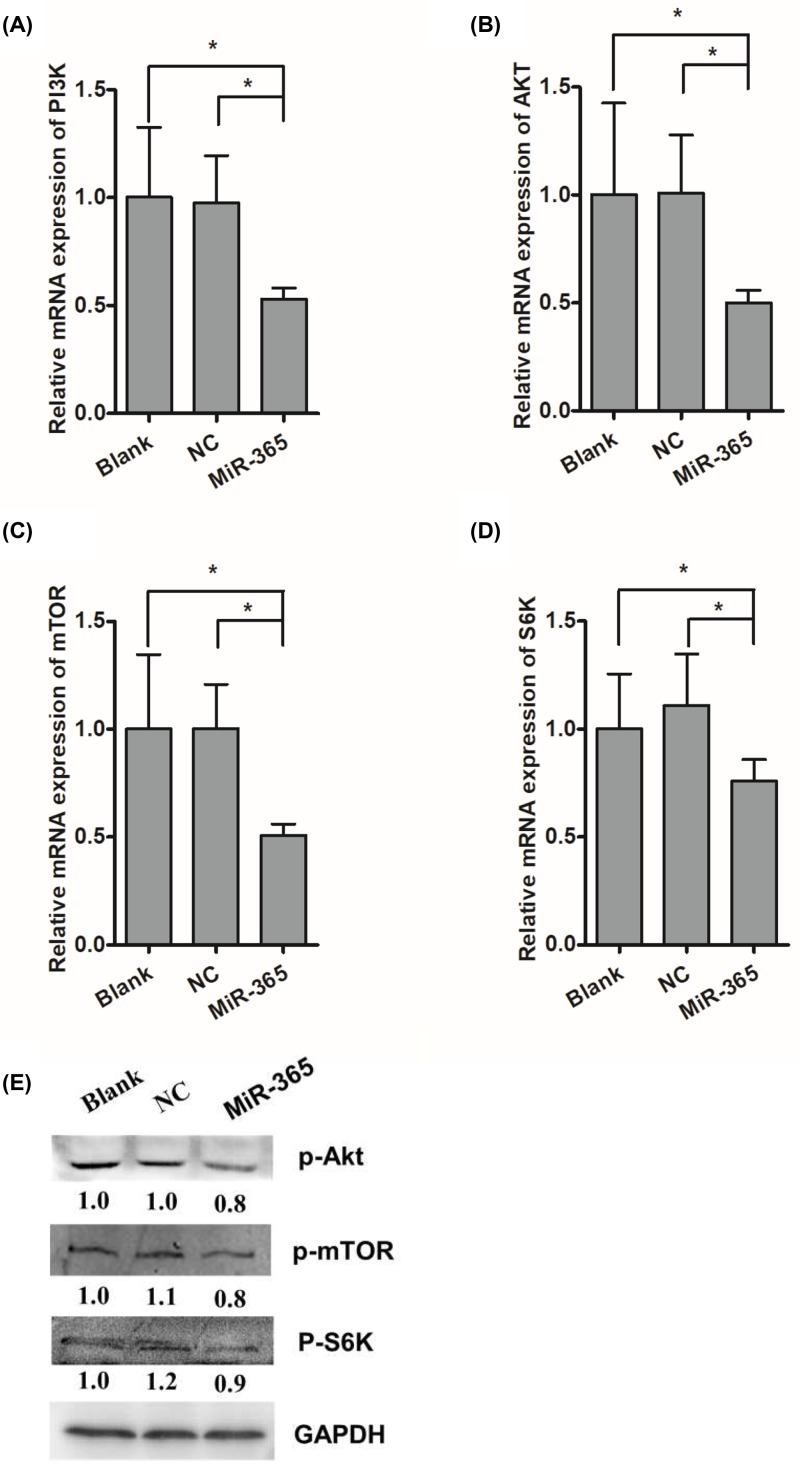
miR-365 down-regulates PI3K/Akt/mTOR/S6K signaling pathway related genes (**A**–**D**) Influences of miR-365 overexpression on the PI3K, Akt, mTOR, S6K mRNA expression. (**E**) Influences of miR-365 overexpression on the p-Akt, p-mTOR, p-S6K protein expression. Each point represents the relative mean ± SD. * denotes significance (*P*<0.05).

Recent study showed that IGF-I promote chicken myoblast proliferation via PI3K/Akt pathway [19]. To further confirm whether miR-365 promotes the duck myoblast proliferation via PI3K/Akt pathway, RT-PCR and Western blot were performed. We found that the level of PI3K, AKT, mTOR and S6K transcripts were significantly down-regulated by miR-365 (Figure 3A–D). Furthermore, the protein level of p-AKT, p-mTOR and p-S6K were also down-regulated by miR-365 (Figure 3E).

### Effects of LY294002 and miR-365 on IGFs/PI3K/Akt/mTOR signaling pathway

**Figure 4:**
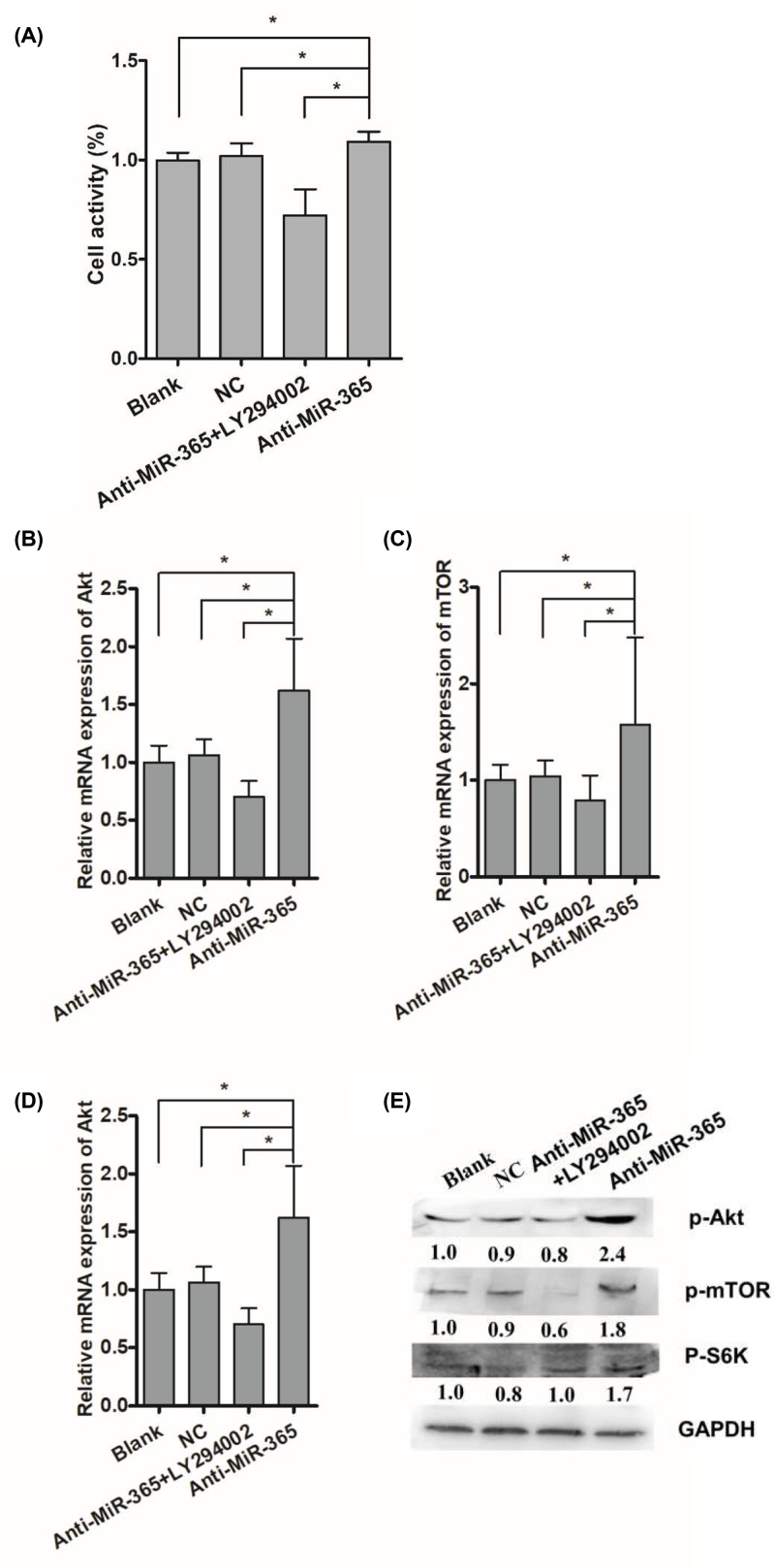
Influences of miR-365 and LY294002 treatment on PI3K/Akt/mTOR/S6K signaling pathway related genes (**A**) CCK-8 assay was performed to detect the cell viability after transfecting with miR-365 and treating with LY294002. (**B**–**D**) Influences of miR-365 overexpression and LY294002 treatment on the Akt, mTOR, S6K mRNA expression. (**E**) Influences of miR-365 overexpression and LY294002 treatment on the p-Akt, p-mTOR, p-S6K protein expression. Each point represents the relative mean ± SD. * denotes significance (*P*<0.05).

Previous study showed that LY294002 could inhibit the protein expression of p-AKT and then inhibit the proliferation of duck myoblast [20]. To verify whether miR-365 inhibit the activation of PI3K/Akt/mTOR pathway, the Akt inhibitor, LY294002 was used. CCK-8 assay revealed that when LY294002 and anti-miR-365 were added together, the cell viability of duck myoblasts significantly decreased to a low level (Figure 4A). Moreover, we showed that the level of PI3K, AKT, mTOR and S6K transcripts were enhanced by anti-miR-365. Importantly, up-regulation of PI3K, AKT, mTOR and S6K by anti-miR-365 were abrogated upon treatment with LY294002 (Figure 4B–D). Moreover, treatment with LY294002 also abolished the up-regulation of p-AKT, p-mTOR and p-S6K protein by anti-miR-365 (Figure 4E).

### Effect of miR-365 on cell cycle regulators

**Figure 5:**
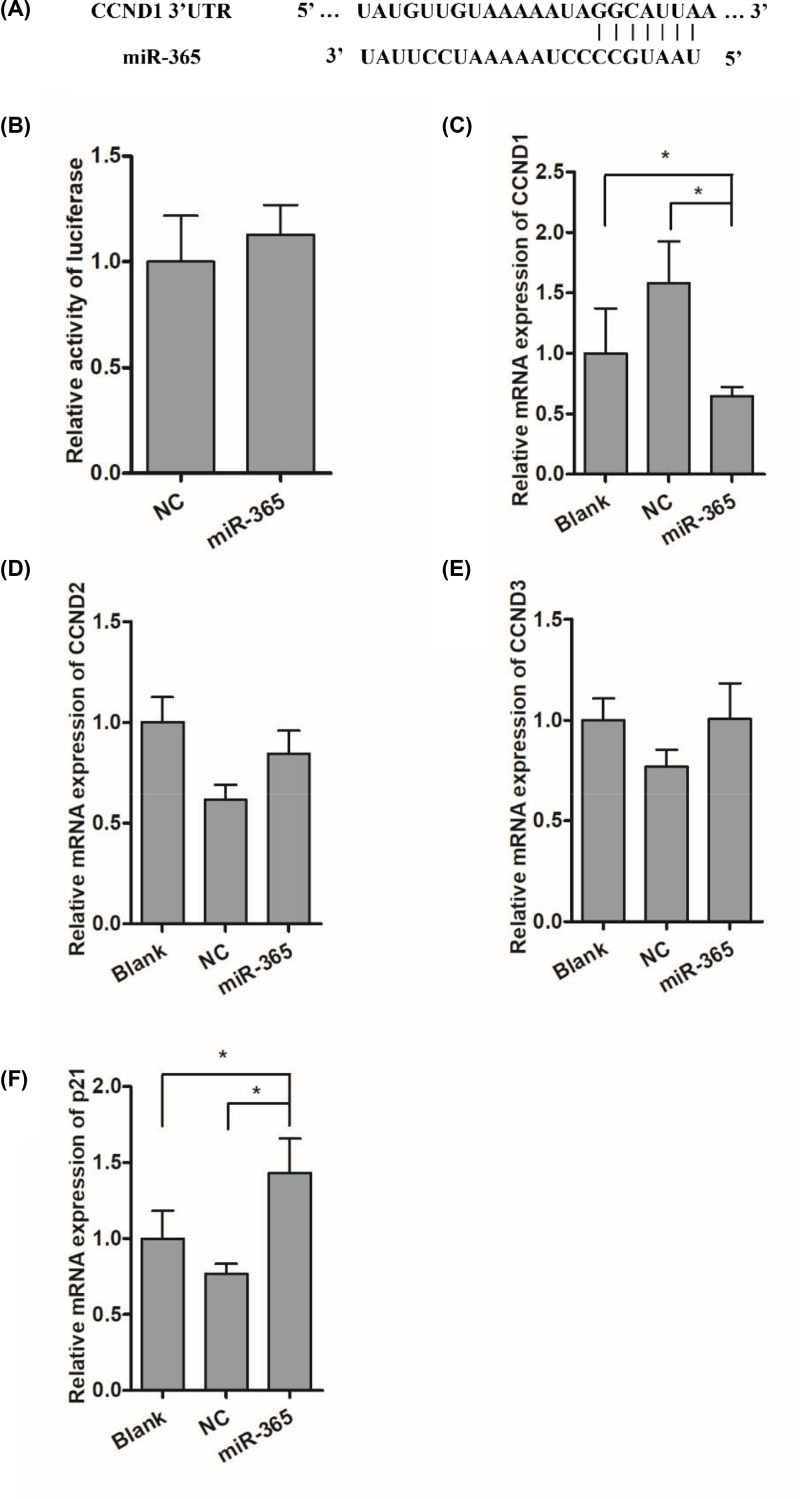
Influences of miR-365 on the family of D-type cyclins (**A**) miR-365 target sequence alignment in the CCND1 3′UTR. (**B**) Activity of a luciferase reporter fused to CCND1 3′UTR fragments transfected into duck myoblast that were kept growing in DMEM. (**C**–**F**) Influences of miR-365 mimic overexpression on CCND1, CCND2, CCND3, p21 expression. Each point represents the relative mean ± SD. * denotes significance (*P*<0.05).

To determine the mechanism by which miR-365 regulate duck myoblast proliferation, the target genes of miR-365 was predicted by using online prediction tool. We showed that CCND1, which is known as an essential cycle regulator, was identified as a candidate gene for miR-365 (Figure 5A). To verify this, the dual-luciferase reporters of CCND1 were co-transfected with miR-365 mimic or control into cells. We found that miR-365 could not significantly alter the firefly luciferase activity of the wild-type CCND1 reporter compared with control group (Figure 5B). Interestingly, the level of CCND1 mRNA was found to be markedly decreased in the miR-365 mimic group (Figure 5C) (*P*<0.05). Except for CCND1, the mRNA level of other cell cycle regulators was examined. We found that miR-365 could not significantly alter the level of CCND2 and CCND3 transcripts (Figure 5D,E), but could markedly increase the level of p21 (Figure 5F) (*P*<0.05).

## Discussion

Growing evidence has demonstrated that muscle-specific miRNAs function as a control center in directing diverse biological processes during myogenic proliferation and differentiation [21–23]. Previous studies showed that miR-365 can remarkably suppress the proliferation of some cancer cells (prognostic significance and anti-proliferation) and promote endothelial cells (ECs) apoptosis (MiRNAs expression in ox-LDL treated HUVECs: miR-365 modulates) [24,25]. In this study, miR-365 significantly inhibits the myoblast cell activity and cell growth, suggest miR-365 can markedly suppresses duck myoblast proliferation.

It is well known that insulin-like growth factors (IGFs) play a central role in regulating skeletal muscle growth by stimulating myoblast proliferation [26,27]. Previous study showed that the time course of increased IGF-I expression is consistent with a role for this growth factor as a mediator of the hypertrophy response, and increased expression of IGF-I in overloaded muscle appears to be stimulating an increase in the DNA content of the muscle [28]. In this study, IGF-I was identified and confirmed as a target gene of miR-365, suggested that miR-365 may suppress duck myoblast proliferation through IGF-I signaling pathways. It has been demonstrated that stimulation of the cellular responses to IGF-I are mediated through the IGF-I receptor via the PI3K/AKT/mTOR pathway [8]. In this study, our results showed that overexpression of miR-365 could significantly decrease the expression of PI3K, AKT, mTOR and S6K at transcript level. Moreover, the expression of p-AKT, p-mTOR and p-S6K also decreased. These results suggested miR-365 suppress duck myoblast proliferation through IGF-1/PI3K/AKT/mTOR/S6K signaling pathways. Previous study showed that LY294002 could inhibit the protein expression of p-AKT and then inhibit the proliferation of duck myoblast [20]. In this study, when LY294002 and anti-miR-365 were added together, the viability of duck myoblasts significantly decreased to a low level, which suggested that LY294002 blocked the positive impact of anti-miR-365 on myoblast viability. Furthermore, LY294002 not only significantly blocked the mRNA expression of Akt, mTOR and p70S6K but also blocked the protein expression of phosphor-Akt, phospho-mTOR (Ser^2448^) and phospho-p70S6K (Ser^417^). These findings suggested that LY294002 block the positive impact of anti-miR-365 on the expression Akt, mTOR and p70S6Kand then block the viability of duck myoblast. In other words, miR-365 could inhibit myoblast proliferation via the a IGF-1/PI3K/AKT/mTOR/S6K signaling pathway.

The proliferation of cells is regulated by cyclins and their associated cyclin-dependent kinases (CDKs). Cyclins represent regulatory subunits that bind, activate and provide substrate specificity for their catalytic partners, CDKs. These cyclin–CDK complexes phosphorylate critical cellular substrates, thereby allowing cell cycle progression [29]. Among all cyclin classes, the family of D-type cyclins (cyclins D1, D2, and D3) stands out as a very unique component of the cell cycle apparatus (Quelle et al., 1993). In the present study, CCND1 was also identified as a potential target gene of miR-365 and it can be significantly inhibited by miR-365, but our result showed miR-365 cannot bind to the CCND1’s 3UTR, suggested that miR-365 can influence CCND1’s expression in an unknown way. It has been demonstrated that Cyclin kinase inhibitors (CKIs) bind to cyclin–CDK complexes, which inactivate the kinase, causing cell cycle arrest and the inhibition of proliferation. There are two families of CKIs, which are classified according to their structural homology and which cyclin–CDK complexes they inhibit. The INK4 family inhibits CDKs active in the G_1_ phase of the cell cycle [30], whereas members of the Cip/Kip family, currently comprising p21CIP1/WAF1 (p21), p27KIP1, and p57KIP2, contain a CDK-binding domain and inhibit both G_1_- and S-phases [31]. In current study, we found that the expression of p21 was markedly increased after miR-365 mimic transfection, suggested miR-365 can influence p21’s expression. Thus, miR-365 may regulate the myoblast proliferation by mediating the expression of cell cycle regulators.

In conclusion, in the present study, we found that overexpression of miR-365 inhibited myoblast proliferation by targeting IGF-I. Moreover, miR-365 decreased the expression of genes involved in the PI3K/Akt/mTOR/S6K pathway. Furthermore, this negative impact of miR-365 on PI3K/Akt/mTOR/S6K pathway can be abrogated by treatment with LY294002. Thus, miR-365 may target IGF-I to inhibit duck myoblast proliferation via PI3K/Akt pathway.

## Abbreviations

ANOVA: analysis of variance
BrdU: 5-bromo-2V-deoxyuridine
CCK-8: cell counting kit-8
CCND1: cyclin D1
CDK: cyclin-dependent kinase
CKI: cyclin kinase inhibitor
DMEM: Dulbecco’s modified Eagle’s medium
FBS: fetal bovine serum
FSHD: facioscapulohumeral muscular dystrophy
IGF-I: insulin-like growth factor-I
miRNA: microRNA
RT-PCR: real-time PCR
S6K: protein S6 kinase
UTR: untranslated region
